# Integrative DNA methylome analysis of pan-cancer biomarkers in cancer discordant monozygotic twin-pairs

**DOI:** 10.1186/s13148-016-0172-y

**Published:** 2016-01-20

**Authors:** Leonie Roos, Jenny van Dongen, Christopher G. Bell, Andrea Burri, Panos Deloukas, Dorret I. Boomsma, Tim D. Spector, Jordana T. Bell

**Affiliations:** Department of Twin Research and Genetic Epidemiology, King’s College London, London, UK; Department of Biological Psychology, VU University Amsterdam, Amsterdam, The Netherlands; MRC Lifecourse Epidemiology Unit, University of Southampton, Southampton, UK; Human Development and Health Academic Unit, Institute of Developmental Sciences, University of Southampton, Southampton, UK; Epigenomic Medicine, Centre for Biological Sciences, Faculty of Environmental and Natural Sciences, University of Southampton, Southampton, UK; Department of Psychology, University of Zurich, Zurich, Switzerland; William Harvey Research Institute, Barts and The London School of Medicine and Dentistry, Queen Mary University of London, London, UK

**Keywords:** DNA methylation, Cancer, Discordant monozygotic twins, Epigenetics, Biomarker, Twin study

## Abstract

**Background:**

A key focus in cancer research is the discovery of biomarkers that accurately diagnose early lesions in non-invasive tissues. Several studies have identified malignancy-associated DNA methylation changes in blood, yet no general cancer biomarker has been identified to date. Here, we explore the potential of blood DNA methylation as a biomarker of pan-cancer (cancer of multiple different origins) in 41 female cancer discordant monozygotic (MZ) twin-pairs sampled before or after diagnosis using the Illumina HumanMethylation450 BeadChip.

**Results:**

We analysed epigenome-wide DNA methylation profiles in 41 cancer discordant MZ twin-pairs with affected individuals diagnosed with tumours at different single primary sites: the breast, cervix, colon, endometrium, thyroid gland, skin (melanoma), ovary, and pancreas. No significant global differences in whole blood DNA methylation profiles were observed. Epigenome-wide analyses identified one novel pan-cancer differentially methylated position at false discovery rate (FDR) threshold of 10 % (cg02444695, *P* = 1.8 × 10^−7^) in an intergenic region 70 kb upstream of the *SASH1* tumour suppressor gene, and three suggestive signals in *COL11A2*, *AXL*, and *LINC00340*. Replication of the four top-ranked signals in an independent sample of nine cancer-discordant MZ twin-pairs showed a similar direction of association at *COL11A2*, *AXL*, and *LINC00340*, and significantly greater methylation discordance at *AXL* compared to 480 healthy concordant MZ twin-pairs. The effects at cg02444695 (near *SASH1*), *COL11A2*, and *LINC00340* were the most promising in biomarker potential because the DNA methylation differences were found to pre-exist in samples obtained prior to diagnosis and were limited to a 5-year period before diagnosis. Gene expression follow-up at the top-ranked signals in 283 healthy individuals showed correlation between blood methylation and gene expression in lymphoblastoid cell lines at *PRL*, and in the skin tissue at *AXL*. A significant enrichment of differential DNA methylation was observed in enhancer regions (*P* = 0.03).

**Conclusions:**

We identified DNA methylation signatures in blood associated with pan-cancer, at or near *SASH1*, *COL11A2*, *AXL*, and *LINC00340*. Three of these signals were present up to 5 years prior to cancer diagnosis, highlighting the potential clinical utility of whole blood DNA methylation analysis in cancer surveillance.

**Electronic supplementary material:**

The online version of this article (doi:10.1186/s13148-016-0172-y) contains supplementary material, which is available to authorized users.

## Background

Despite global research efforts, cancer remains one of the leading causes of death in economically developed countries, second only to cardiovascular disease [1, 2]. Early and accurate detection greatly increases the odds of successful treatment. Furthermore, considering the worldwide expected increase in cancer incidence [3], the need for new cost-effective detection or prediction methods to improve disease outcome, such as accurate and precise biomarkers is paramount. One key area of focus is the development of cancer biomarkers in non-invasive tissues, such as peripheral blood or serum, which can accurately diagnose early lesions, and hence, improve survival, and even identify individuals at risk [4–6].

DNA methylation is a molecular mark that has a great potential as biomarker for early cancer detection in non-invasive tissues. It is a relatively stable epigenetic mark that can be influenced by DNA sequence variation, but also environmental factors and stochastic changes that occur over a lifetime [7–11]. It provides a potential link between environmental conditions and exposures with changes in gene activity either directly or in combination with genetic susceptibility by influencing penetrance and expressivity [12]. Aberrant DNA methylation is associated with a broad range of diseases [13], age [14], environmental factors such as smoking [8, 15–18], and is especially prevalent in human cancer tissues [19, 20]. Epigenetic changes that occur in carcinogenesis can be detected in early neoplastic tissues, as well as tumour-derived DNA in plasma or serum of patients [21].

A complex interaction of environmental factors, stochastic events, and genetic susceptibility can lead to cancer development. Blood samples are known to reflect the health status of an individual and evaluating whole blood or blood cell types in particular, might reveal specific or systemic changes in the host that are associated with malignant disease. Indeed, DNA methylation signatures in blood have been associated with cancerous and pre-cancerous primary locations such as the breast [22, 23], colon [24], bladder [25], and ovary [26]. To the best of our knowledge, no study to date has attempted to identify pan-cancer epigenetic biomarkers, that is, epigenetic biomarkers indicative of cancer of multiple different origins, in whole blood samples. However, pan-cancer analyses have been conducted directly in tumour tissues, for example, the Cancer Genome Atlas Research Network launched a pan-cancer project in 2012 [27], and other recent studies that have identified pan-cancer DNA methylation patterns in different tumour tissues [28, 29]. The identification of a blood-based DNA methylation biomarker that can predict cancer or pan-cancer development would be a highly valuable asset to the current screening processes, as well as contributing to understanding potential common systemic changes associated with disease.

The aim of the present study was to explore whole blood DNA methylation patterns in cancer-discordant female monozygotic (MZ) twin-pairs to identify pan-cancer-associated epigenetic changes. MZ twins are matched for age, sex, cohort effects, many maternal influences, and early environment factors, and have nearly identical genomes. Discordant MZ studies are therefore a particularly powerful and less biased design for detecting disease-related epigenetic differences [30]. In the current study, we analysed blood samples that were taken from up to 11 years before or up to 5 years after cancer diagnosis, which allowed us to explore biomarker stability over time. This is the first study in MZ twin-pairs to explore pan-cancer-associated blood DNA methylation changes with a focus on the detection of early neoplastic development. Our results identify four CpG sites that are associated with cancer status, with follow-up replication and gene expression analyses, and highlight signals with promising biomarker potential.

## Results

### Global methylation profiles in cancer-discordant monozygotic twin-pairs

**Fig. 1:**
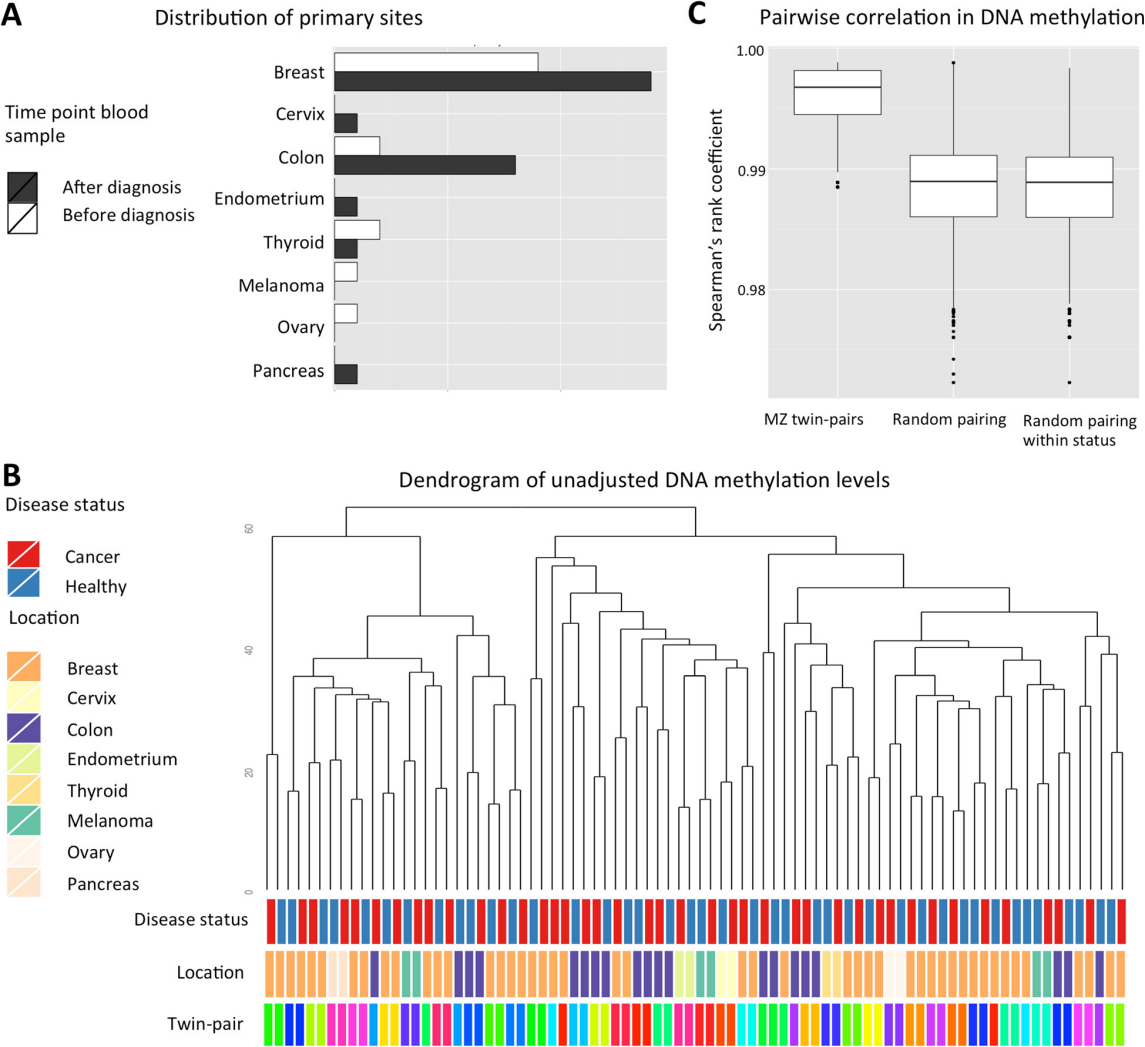
Diagnostic characteristics and global methylation profiles of 41 cancer-discordant MZ twin-pairs. **a** Number of cases for each primary location of cancer, where a blood sample was obtained before (*white*) and after (*black*) cancer diagnosis. **b** Dendrogram of the unadjusted global methylation profiles. Annotation bars denote each individual’s cancer status, type of cancer (identical for both twins in a pair), and family identifier (identical for both twins in a pair). **c** Pair-wise correlation in DNA methylation profiles shows greater similarity within MZ pairs, compared to pairs of unrelated individuals, either paired at random or at within affection status

We analysed genome-wide DNA methylation profiles in whole blood samples of 41 female monozygotic twin-pairs discordant for cancers of the solid organs. Affected individuals in this sample had cancers at various primary sites: the breast, cervix, colon, endometrium, thyroid gland, skin (melanoma), ovary, and pancreas (Fig. 1a). To assess global DNA methylation variation, unsupervised hierarchical clustering of unadjusted normalised DNA methylation values was performed. Thirty-five of the 41 MZ twin-pairs (85.4 %) clustered with their co-twin (Fig. 1b). The remaining MZ twin-pairs were clustered by array, underlining the importance of correcting for technical covariates in downstream analyses. Subsequently, the top 1000 probes with the highest standard deviations were assessed with unsupervised hierarchical clustering, to determine if the most variable CpG sites combined were associated with cancer status. All MZ twin-pairs now clustered together and confirmed that MZ twin-pairs were globally more similar to each other, compared to unrelated individuals with the same affection status (cancer vs. non-cancer). Furthermore, MZ twin-pairs showed high within-pair correlations in normalised unadjusted DNA methylation values (mean Spearman’s rank correlation coefficient (*r*_S_) = 0.986), which was significantly greater than pairing at random, or pairing at random by affection status (Fig. 1c, *P* = 2.2 × 10^−16^). The high within-pair correlation is comparable to genome-wide average correlations estimated in newborn twins, ranging from 0.98 to 0.99 for the placenta, cord blood mononuclear cells, and human umbilical vascular endothelial cells [31]. A similar average correlation is seen in peripheral blood at 15 years (0.99) [32] and middle-aged individuals (0.98) [33] as well as in the adipose tissue of the middle-aged individuals (0.992) [34].

### Pan-cancer-associated differentially methylated positions

**Fig. 2:**
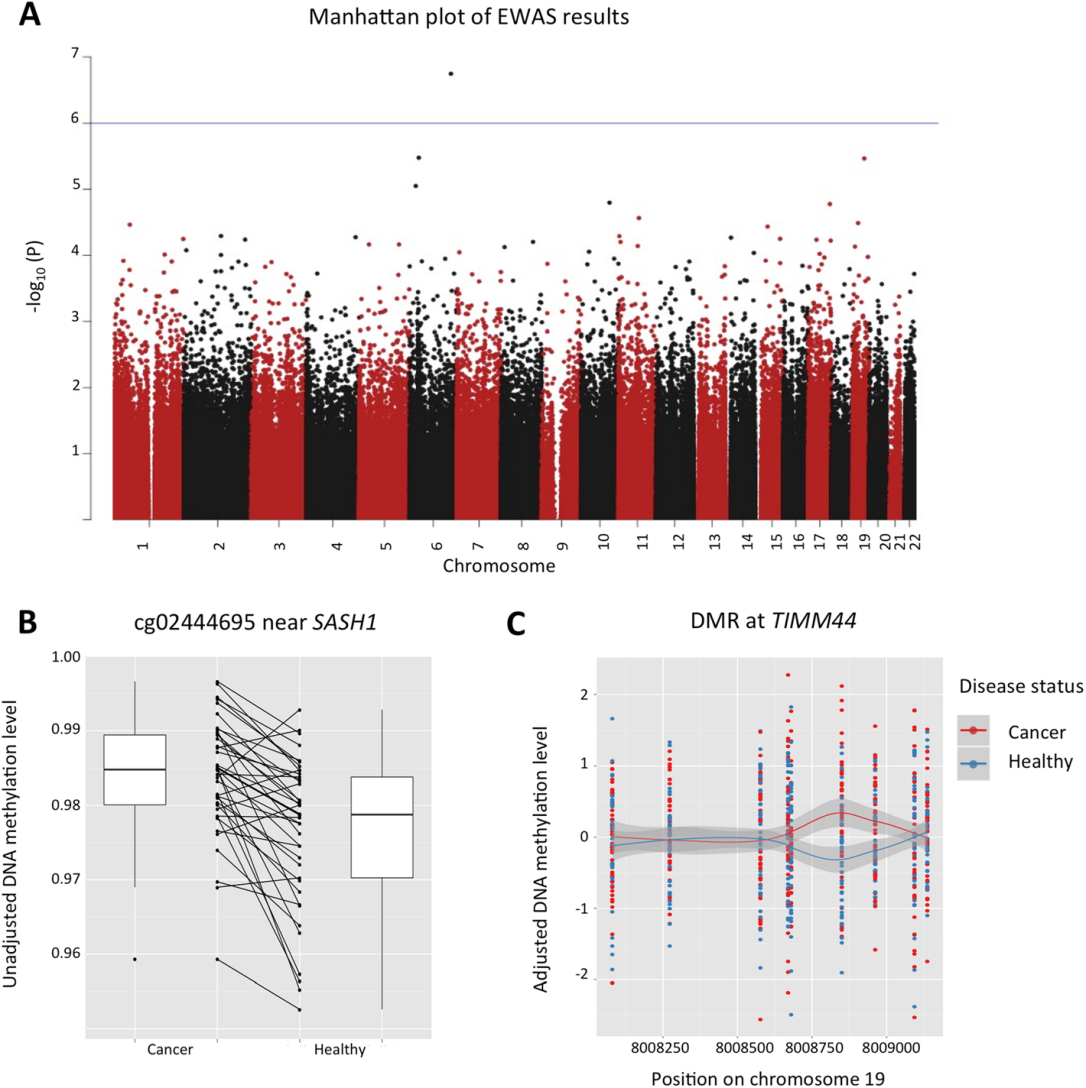
Pan-cancer epigenome-wide results in 41 discordant MZ twin-pairs. **a** Manhattan plot of the epigenome-wide association results in 41 pan-cancer-discordant MZ twin-pairs, where each point represents the observed −log_10_ *P* value at a CpG-site. **b** Direction of association at the top-ranked signal cg02444695, near *SASH1*. Results are plotted using normalised unadjusted beta values of cancer-affected individuals (*left*) and healthy individuals (*right*). The *lines* connect co-twins in twin-pairs and indicate a consistent direction of the effect with an average of 0.7 % within twin-pairs with a range of 0.9 to 3.0 %. The three suggestive probes are included in Additional file 1. **c** Pan-cancer DMR at *TIMM44*. Results are plotted using adjusted DNA methylation values at each CpG site in the DMR for individuals affected by cancer (*red*) and healthy co-twins (*blue*). *Smooth* (LOESS) *lines* with standard error are plotted for both groups. The CpG site driving the signal is at chr19:8,008,850 (hg19)

Differences in DNA methylation levels were next analysed at single CpG sites across the genome within 41 female MZ twin-pairs discordant for cancer development. DNA methylation values were adjusted for technical and biological covariates by using the first five principle components that explained 46 % of variance in the data. The first five principle components were significantly associated with variables that included technical covariates (batch and array), blood cell type composition, but not cancer status (see “Methods” section). The epigenome-wide association scan (EWAS) analysis identified one novel pan-cancer differentially methylated position (DMP) at a false discovery rate (FDR) threshold of 0.10 for probe cg02444695 (*P* = 1.8 × 10^−7^) located in an intergenic region. Additionally, three suggestive pan-cancer DMPs (*P* < 1.0 × 10^−5^) were identified for probes cg26079695 in *COL11A2*, cg27094856 in *AXL*, and cg21046959 in *LINC00340* (Table 1, Fig. 2a).

**Table 1:** Top-ranked results from EWAS of 41 cancer discordant MZ twin-pairs

				Discovery EWAS *N* = 41 MZ twin-pairs (*prior to and after diagnosis*)			Replication *N* = 9 MZ twin-pairs (*NTR replication*)		Discordant *vs* healthy MZ pairs *N* = 9 *vs* *N* = 480 (*NTR variability*)	EWAS prior to diagnosis *N* = 15 MZ twin-pairs (*prior to diagnosis only*)		
CpG	Position (hg19)	Associated gene	Location	Rank EWAS	Mean difference*	*P* value	Mean difference*	*P* value	*P* value	Rank EWAS	Mean difference*	*P* value
cg02444695	Chr6:148950185	–	–	1	0.70	1.80 × 10^−7^	−0.64	0.26	0.09	10	0.88	2.40 × 10^−5^
cg26079695	Chr6:33143273	*COL11A2*	Intron	2	−0.67	3.32 × 10^−6^	−050	0.23	0.34	1518	−0.88	4.10 × 10^-3^
cg27094856	Chr19:41732589	*AXL*	Intron	3	0.56	3.41 × 10^−6^	0.02	0.96	0.05	3801	0.51	9.71 × 10^−3^
cg21046959	Chr6:22180833	*LINC00340*	Transcript	4	−0.53	8.89 × 10^−6^	−0.43	0.37	–	407	−0.73	1.21 × 10^-3^

*The mean differences are determined using adjusted DNA methylation values and calculated as cancer − unaffected twin

The most associated pan-cancer DMP, cg02444695, exhibited consistently higher DNA methylation levels in cancer-affected twins compared to the healthy co-twins (Fig. 2b). The CpG-site falls in an intergenic region, 70 kb upstream of the nearest gene, *SASH1*, a known tumour suppressor gene previously associated with aggressive tumour growth and metastasis formation in different types of cancer. Of the three genome-wide suggestive probes (Table 1, Additional file 1), cg27094856 was located in the fourth intron of the *AXL* gene, which is implicated in many cancers and is a therapeutic target for antibody-based therapies [35–37]. Another suggestive signal was obtained at cg21046959 directly in *LINC00340*, and the *LINC00340* transcript has been linked to both neuroblastoma and ovarian tumours [38, 39].

We pursued replication at the four top-ranked results in an independent twin sample from the Netherlands Twin Registry (NTR). First, we analysed nine cancer-discordant MZ twin-pairs from NTR with the same analysis pipeline used for the discovery findings. We observed a similar direction of association at the probes, except for cg02444695 (Table 1, Additional file 2), but no results surpassed nominal significance in the replication sample, which may be due to the very small sample size. Second, we assessed how much variation in DNA methylation occurs at the top-ranked CpG sites in a healthy population. We expect if the observed changes are not stochastic effects and due to cancer status, to see less variation at these sites in a healthy population than cancer-discordant twin-pairs. To this end, data for the three of the four top-ranked probes were available for both the nine cancer-discordant MZ twin-pairs and 480 healthy MZ twin-pairs from NTR. We compared the absolute within-pair differences in DNA methylation at cg02444695, cg26079695, and cg27094856 (Table 1, Figures in Additional file 3). We observed greater within-pair difference in DNA methylation in cancer-discordant twins compared to healthy twins, with nominally significant effects at cg27094856 in *AXL* (healthy median 0.78 % vs. cancer median 1.44 %, *P* = 0.047), and near significant effects at cg02444695 near *SASH1* (healthy median 1.48 % vs. cancer median 2.32 %, *P* = 0.091).

### Pan-cancer-associated differentially methylated regions

We next aimed to identify potential pan-cancer DMRs, that is, larger genomic regions containing multiple CpG-sites that exhibit consistently different DNA methylation levels between the discordant MZ twin-pairs. We applied the ‘bump hunting’ method [40] to define DMRs with few modifications to account for twin structure present in our dataset. We kept the paired structure in the data and used the differences in PC-adjusted DNA methylation values, as described above, per twin-pair in the peak-calling algorithm. One DMR was identified on chromosome 19:8,008,080-8,009,137 (hg19) spanning ~1 kb (*P* = 0.01, Fig. 2c). The DMR is mainly driven by a single CpG (chromosome 19:8,008,850) ranked 24th in the single-CpG EWAS and its two adjacent CpGs that are hypermethylated in cancer-affected twins. This region overlaps a 5′ CpG island within an active promoter across multiple tissue types (according to ChromHMM), approximately ~1500 bp from the transcription start site (TSS) of *TIMM44. TIMM44* has previously been associated with familial non-medullary thyroid carcinoma [41], aggressive serous ovarian cancers [42] and breast cancer recurrence [43].

### No enrichment for cancer risk factors smoking and age

Enrichment for the two major risk factors for cancer development, age and smoking, was assessed in the 500 top-ranked pan-cancer DMP CpG sites. We obtained previously published age and smoking DMPs in whole blood, and assessed whether these CpG-sites tended to co-occur with the pan-cancer DMPs identified in the 41 discordant MZ pairs. The first five PCs used to adjust DNA methylation levels in the EWAS were not significantly associated with either smoking or age; however, they could account for some of the variation observed of these variables. Enrichment for age DMPs was assessed using the results of Steegenga et al. [44] that combined eight studies totalling 7318 age DMPs that were also available in our genome-wide dataset. There was no enrichment (eight probes, *P* = 1) within the top 500 ranked probes for known age DMPs, as compared to the remaining probes genome wide. To assess for enrichment of smoking signals, we used smoking DMP results from the largest whole blood DNA methylation study to date using the HumanMethylation450 BeadChip by Zeilinger et al. [8]. There were 948 smoking DMPs that were also available in our genome-wide dataset, and we did not observe an enrichment of smoking DMPs in the top 500 ranked EWAS associations (three probes, *P* = 0.089). None of the four top-ranked probes had been previously associated with age or smoking status. Finally, no significant enrichment was observed in the top 500 results from an EWAS performed correcting only for batch effects and estimated cell counts (see “Methods” section). Taken together, the pan-cancer DMPs seem indicative of a more complex representation of the risk factors or disease biology.

### Biomarker potential: methylation analysis in samples obtained prior to diagnosis

**Fig. 3:**
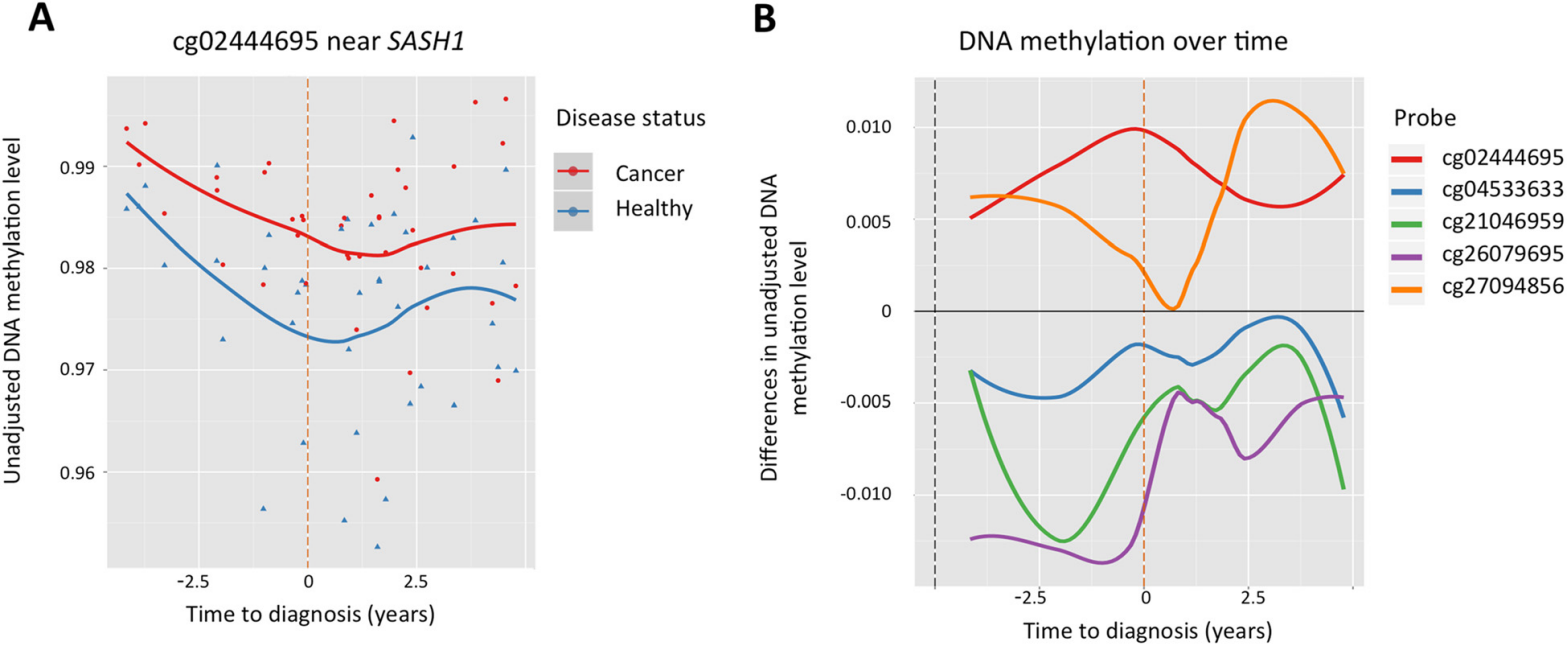
Differential methylation with respect to time of cancer diagnosis. **a** Unadjusted DNA methylation values at cg02444695 (near *SASH1*) in affected individuals (*red*) and healthy co-twins (*blue*), shown with respect to time of diagnosis (years) with *smooth* (LOESS) *lines* fitted for both groups. The *orange vertical line* represents the time of diagnosis. **b** Methylation differences within twin pairs at the four top-ranked DMPs and cg04533633 (at *COX7C*). Each *smooth* (LOESS) *line* represents the methylation difference (affected − unaffected twin) at an individual probe (see legend)

To identify pan-cancer DMPs that could serve as biomarkers for early diagnosis, we performed analyses taking into account the time of cancer diagnosis. We selected a subset of 15 discordant MZ twin-pairs from the 41 female MZ twin-pairs where blood samples were obtained prior to date of official cancer diagnosis, in a 0–5-year period preceding diagnosis (Fig. 1a). EWAS in the 15 MZ pairs was performed using the same approach as the analyses in the 41 MZ twin pairs, and the top-ranked results included signals in the promoters (within 200 bp of the TSS) of genes *COX7C* and *U2AF1* that have been previously linked to cancer (Table provided in Additional file 4). Specifically, the second ranked association in *COX7C* was located in a region previously identified as one of the nine loci that most significantly associated with bladder cancer in whole blood samples [45], and in both analyses, hyper-methylated effects were observed in cancer-affected individuals. In a pan-cancer tumour tissue analysis, recurrent somatic mutations identified in *U2AF1* were shown to induce splicing inducing transcriptome changes [46]. Our previous most-associated pan-cancer DMP (cg02444695 near *SASH1* in 41 MZ pairs) remained strongly significant (*P* = 2.40 × 10^−5^) and with the same direction of association in the new EWAS prior to cancer diagnosis, and was now ranked tenth overall (Table 1, Fig. 3a). The suggestive probes from the original 41 MZ pair EWAS also remained nominally significant in the new analysis and in the same direction of association (Table 1), and in the majority of cases (cg02444695, cg26079695, cg21046959), greater differences between MZ twin-pairs were identified in the samples collected before diagnosis.

### Pan-cancer-associated biomarker stability over time

We next explored the relationship between the time of blood sample extraction and the observed DNA methylation differences in cancer-discordant twins at the four top-ranked pan-cancer DMPs and the pan-cancer DMP located at *COX7C* identified above in more depth. The greatest DNA methylation difference at the top-ranked probe (cg02444695 near *SASH1*) between the MZ twin-pairs was observed when the DNA sample was obtained earlier in the same year as cancer diagnosis (Fig. 3b). The second- (cg26079695 in *COL11A2*) and fourth- (cg21046959 in *LINC00340*) ranked probes displayed greatest effects in the 5-year period prior to official diagnosis whereas the third probe (cg27094856 in *AXL*) showed the largest differences in the 5-year period after official diagnosis. There was no significant correlation between time to diagnosis and age at blood sample collection (*P* = 0.29, Additional file 5: Figure S1A); therefore, these results cannot be explained by the effect chronological age. The top-ranked probe shows differential methylation across all ages included in the large sample (Additional file 5: Figure S1B).

The observed early DNA methylation change between MZ twin-pairs was further explored for the top-ranked probes by including five additional cancer-discordant MZ twin-pairs where blood samples were obtained between 5 and 11 years prior to cancer diagnosis (Additional file 5: Figure S1C–F). At the top-ranked probe (cg02444695), the DNA methylation levels show a reverse pattern prior to 5 years to the diagnosis, the time window of the main study (Additional file 5: Figure S1C). Therefore, we conclude that DNA methylation differences at cg02444695 do not arise from treatment, as these changes can be observed in individuals specifically up to 5 years prior to cancer diagnosis.

### Functional follow-up of pan-cancer differential methylation results

**Fig. 4:**
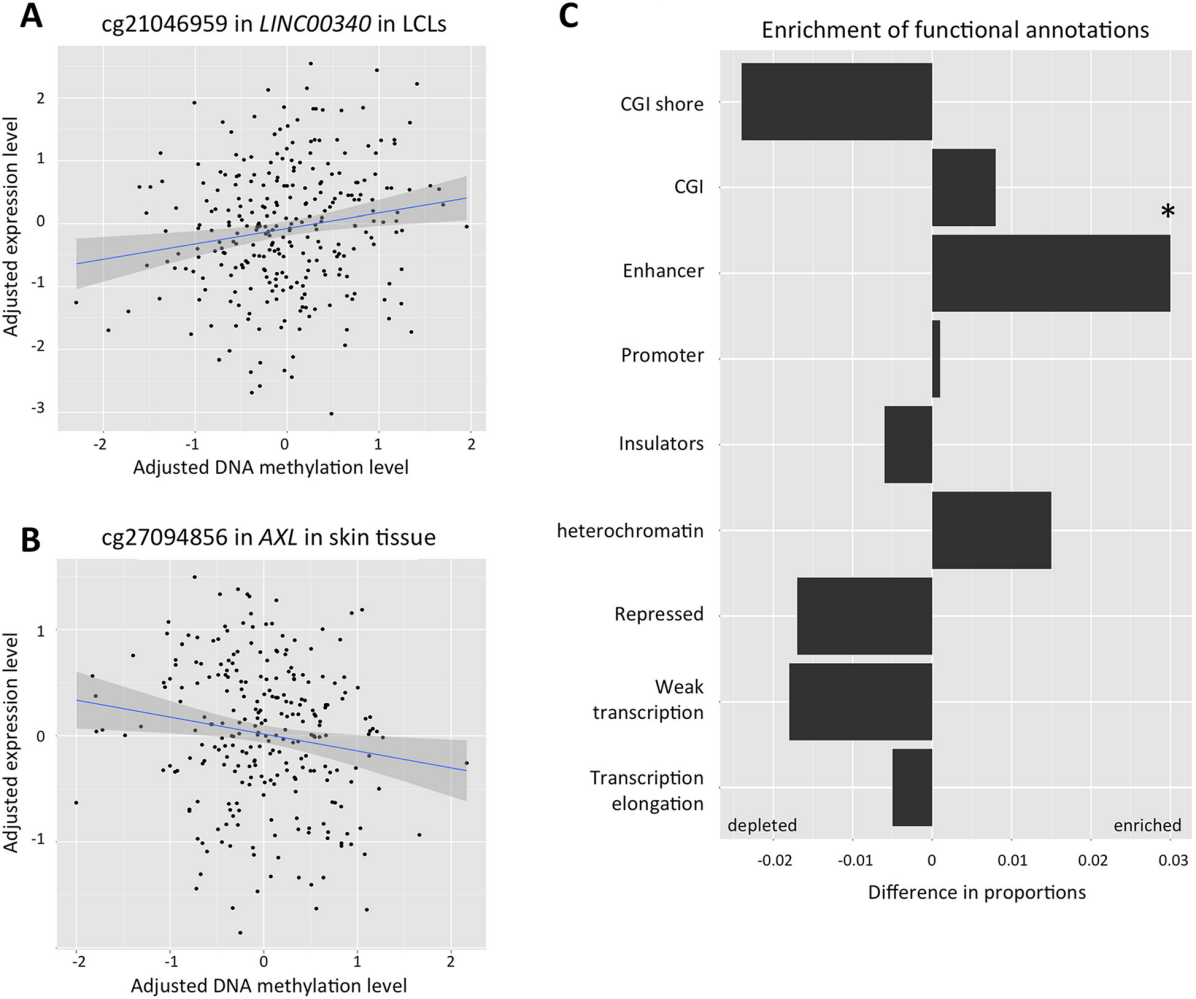
Functional follow-up of top-ranked pan-cancer DMPs. Adjusted whole blood DNA methylation profiles compared to adjusted gene expression levels for (**a**) cg21046959 in blood and ILMN_1809352 (*PRL)* in LCLs, and (**b**) cg27094856 in blood and ILMN_1701877 (*AXL)* in skin. Data are obtained from 283 healthy middle-aged females and lines represent the least squares regression fit. **c** Enrichment analysis of genomic annotation categories within the 500 top-ranked cancer DMPs. The bars indicate the difference in proportion of DMPs compared to the remainder of probes used in the study in the corresponding genomic annotation class. Nominally significant results were obtained for the ‘enhancer’ category (*P* = 0.03)

The four top-ranked pan-cancer DMPs and the pan-cancer DMR were first explored for association with gene expression levels of the closest available transcripts. Analyses were performed in 283 healthy individuals for whom both whole blood DNA methylation levels and gene expression profiles from the multiple tissues (the lymphoblastoid cell lines (LCL), skin, and adipose) were available. We identified two nominally significant correlations of DNA methylation at cg21046959 with LCL gene expression of the closest protein-coding transcript of *PRL*, located ~100 kb upstream of the CpG-site (*r* = 0.17, *P* = 4.5 × 10^−3^), and at cg27094856 with skin tissue expression levels of *AXL* (*r* = −0.15, *P* = 0.01) (Fig. 4a, b). We did not observe these correlations in the other available tissues (Table 2). ENCODE annotation data identified the CpG correlated to expression of *PRL* to be located in a heterochromatin block in the GM12878 B-lymphocyte cell line, although within an active promoter and weak enhancer in human embryonic stem cell line (H1-hESC) and leukaemia cell line (K562), respectively. The CpG site negatively associated with *AXL* expression levels in the skin tissue is identified by ENCODE to be located in a strong enhancer in epidermal keratinocytes and inactive or poised promoter in the GM12878 B-lymphocyte cell line. Higher expression levels of *AXL*, as described previously are implicated in proliferation, migration, and resistance to therapy of many cancers [35–37].

**Table 2:** Gene expression analysis in of top ranked probes from EWAS in 283 healthy individuals

						LCL cell lines		Adipose tissue		Skin tissue	
CpG	Associated gene	Location	Nearest gene expression transcript probe	Name	CpG distance	*r*	*P* value	*r*	*P* value	*r*	*P* value
cg02444695	–	–	ILMN_2185984	*SASH1*	77 kb upstream	−0.02	0.79	0.01	0.91	0.03	0.59
cg26079695	*COL11A2*	Intron	ILMN_2311456	*COL11A2*	–	0.04	0.46	0.07	0.21	0.09	0.17
cg27094856	*AXL*	Intron	ILMN_1701877	*AXL*	–	0.01	0.91	0.07	0.20	−0.15	0.01
cg21046959	*LINC00340*	Transcript	ILMN_1809352	*PRL*	106 kb downstream	0.17	4.5 × 10^−3^	0.03	0.59	0.02	0.74
cg14044916	*TIMM44*	TSS1500	ILMN_1784031	*TIMM44*	–	0.02	0.75	0.05	0.44	0.04	0.55

We then tested for enrichment of functional annotations within the 500 top-ranked pan-cancer DMPs compared to the remaining CpG sites used within the study, hypothesising that this analysis could reveal systemic changes in the body associated with malignant tumour development (Fig. 4c). A nominally significant enrichment (*P* = 0.03) was observed for enhancers (pooled data from ENCODE) and driven by state 7 “weak enhancer” ChromHMM category (*P* = 0.03). In addition, we observed weak non-significant depletion of differential methylation in CGI shores, repressed regions, and weakly transcribed regions.

## Discussion

This study examined MZ twin pairs discordant for multiple types of cancer to identify a pan-cancer DNA methylation signature in peripheral blood independent of host genetic variation. We identified one novel epigenome-wide significant pan-cancer DMP at a FDR threshold of 10 %, located in an intergenic region upstream of a known tumour suppressor gene *SASH1*, and three suggestive pan-cancer-associated signals in the genes *COL11A2* and *AXL*, and in *LINC00340*, two of which have previously been linked to cancer (*AXL*, *LINC00340*). In a subset analysis of whole blood samples obtained before cancer diagnosis, we identified a signal in the promoter of *COX7C*, at the same site where whole blood DNA methylation was previously associated with bladder cancer [45]. We also considered regional-based DNA methylation changes, and observed one potential pan-cancer DMR in the *TIMM44* gene, which was also previously linked to cancer. Despite cancer discordance, we did not observe global differences in DNA methylation profiles and found that MZ twin-pairs exhibited greater within-pair correlation than random pairing of individuals, as previously reported in healthy twins [31–34, 47].

The peak genome-wide significant pan-cancer DMP at cg02444695 upstream of the *SASH1* tumour suppressor gene, showed consistently higher DNA methylation in the cancer-affected compared to their healthy co-twins, on average 0.7 % (range −0.9 to 3.0 % in normalised unadjusted DNA methylation levels). *SASH1* expression has been negatively associated with aggressive tumour growth and metastasis formation in different types of cancer, including the breast, colon, and bone [48–50]. Annotation data from the ENCODE project identified the region harbouring cg02444695 as a weak transcribed region proximal (~500 bp) to a weak/poised enhancer, based on ChromHMM in the GM12878 B-lymphocyte cell line (LCL) which is biologically closest to our whole blood sample. Consistent with this, we observed a weak negative correlation between whole blood DNA methylation and LCL *SASH1* expression (see section Gene expression analysis of top ranked probes, Table 2). However, Epstein-Barr virus (EBV) transformation for LCLs impacts DNA methylation profiles [51] and results in less variability in gene expression in mature LCLs [52]. Direct comparison between whole blood and LCL gene expression has revealed these sources of variation to be distinct, although LCL expression changes have been associated with phenotypes such as smoking [53]. Although we could not directly replicate the differential effect at cg02444695, this may be due to the small replication sample size of nine cancer-discordant MZ twin-pairs. However, we observed near significant greater blood methylation variability at this site in cancer-discordant twins compared to healthy concordant twins. The DNA methylation differences observed at this most associated pan-cancer DMP persisted in samples that were obtained prior to cancer diagnosis, indicating that this signal is not driven by cancer treatment and can be detected prior to or during early tumour development and could represent accrued environmental risk factor exposures, common systemic effects due to the presence of the tumour, or surrogate changes of the tumour. The effect is particularly strong in a critical time window of a maximum of 5 years prior to diagnosis, which makes it a very promising blood-based biomarker candidate.

Two of the genome-wide suggestive pan-cancer DMPs were also located in cancer-related gene *AXL* and lncRNA *LINC00340*. Cg27094856 was located in the fourth intron of the *AXL* gene, which is implicated in proliferation, migration, and resistance to therapy of many cancers and is a therapeutic target for antibody-based therapies [35–37]. Hypermethylation was observed in twins affected with cancer, and this effect was also observed in the replication sample of nine MZ twin-pairs, although it did not surpass nominal significance in this small sample. Furthermore, similar to the results for cg02444695, there was significantly greater blood methylation variability at cg27094856 in cancer-discordant twins compared to healthy concordant twins. A negative correlation was observed between DNA methylation at cg27094856 and the protein-coding transcript of *AXL* in the skin tissue only. Higher expression levels of *AXL* are found in many cancers tissues and implicated in proliferation, migration, and therapy resistance [35–37]. However, we did not observe a correlation in *LCL*s suggesting that this could be a biomarker in whole blood for common systemic effects. Exploring differential methylation results at this site with respect to time of diagnosis suggests that the majority of differential methylation arises in the samples after cancer diagnosis; therefore the methylation effect may be as a result of cancer treatment, systemic immune response to the presence of the tumour, or the tumour itself.

The second suggestive signal in a cancer-related gene was obtained at cg21046959 in *LINC00340*, and showed hypomethylation in cancer-affected twins. This *LINC00340* transcript has been identified as a neuroblastoma susceptibility gene and was shown to be hypermethylated within its promoter in clear cell ovarian tumours [38, 39]. This is consistent with the observed direction of association in our results, as gene body hypomethylation and promoter hypermethylation are both associated with decreased expression [54]. We explored DNA methylation with the available gene expression data, but unfortunately, transcriptomic data was not available for *LINC00340*. However, we identified a positive correlation between DNA methylation at cg21046959 in whole blood and gene expression of *PRL* in LCL cell lines, but not in the skin or adipose tissue in 283 healthy female individuals. Previously, greater expression of *PRL* has been associated with progression of tumour development in different cancers [55–57]. Differential methylation effects at this locus were greatest in twin pairs sampled 2 years (range 0–5) prior to cancer diagnosis, and minimal after cancer diagnosis, suggesting that this locus also is of biomarker potential. The third genome-wide suggestive pan-cancer DMP was located in the *COL11A2* gene, which to our knowledge has not been linked to cancer. This site also exhibited the greatest differential methylation effects within 0–5 years before diagnosis, therefore suggestive of biomarker potential.

Analysis of blood samples preceding diagnosis identified a signal in the promoter of the *COX7C* gene. The same site was previously associated with bladder cancer in whole blood samples [45]. Interestingly, none of the twins included in this study were diagnosed with cancer of the bladder as of yet. This suggests that the common observed effects prior to cancer diagnosis could include bladder cancer as well and requires further follow-up.

In addition to single-CpG-based analyses, we also considered differential methylation effects that spanned larger genomic regions. The genome-wide DMR analysis had attenuated findings, but highlighted a top-ranked pan-cancer DMR in the promoter of the *TIMM44* gene. Germline genetic variants in this gene have been associated with familial non-medullary thyroid carcinoma [41]. Furthermore, an intragenic CpG island in *TIMM44* has been found to be hypomethylated in aggressive serous ovarian cancers [42], and its expression is positively associated with recurrence after chemotherapy in breast cancer patients [43]. Given its implication in multiple cancers, this region requires further follow-up with higher resolution technologies.

To our knowledge, this is the first study to explore pan-cancer blood biomarkers in cancer-discordant MZ twin-pairs. One of the limitations of our study is that cancer is a heterogeneous disease with differing aetiology across many tissue types. Therefore, considering multiple types of cancer could potentially dilute cancer type-specific effects and may reduce power to detect true associations. However, pan-cancer signatures have previously been identified in tumour tissues spanning changes in DNA methylation as well as the proteome, somatic mutations, and somatic copy-number alterations [28, 58–61]. These findings show that shared signals across different tumour types can occur, and that these may also be associated with common systemic effects or surrogate changes.

The primary aim of our study was to identify blood-based pan-cancer biomarkers. These effects may capture either a general systemic (immune) response of the body to tumour development, accrued environmental risk exposures leading to cancer development, or changes that are present across cell types. A limitation is that although blood is an ideal sample for non-invasive biomarker screening, it is a surrogate tissue with a heterogeneous cell population. Here, we addressed cellular heterogeneity by correcting for biological covariates that capture the proportion of major white blood cell fractions. Our analyses of blood-based pan-cancer biomarkers detect signals in known tumour-associated genes, and extend previous findings of pan-cancer DNA methylation signatures to blood. Although we corrected for cellular heterogeneity, the identified signals may also reflect minor immune cell fractions or rare cell subtypes that are not covered by the applied cell-type correction, as shown for the *GPR15* smoking findings in whole blood samples [62]. Further research is needed to investigate if these signals persist in sorted blood cell types, in the tissue of the primary tumour site, and tumour tissue itself, for example, to identify if these changes are present across cell types and could be surrogate changes of the tumour internal environment. Future studies are also needed to explore the longitudinal stability of these changes in different cell types over time.

By using a discordant MZ twin design, our aim was to identify systemic epigenetic changes that are independent of genotype or early environment. On the other hand, recent publications have identified genetic variants associated with DNA methylation (methylation quantitative trait loci), and these could potentially also interact with the environment. Future studies in larger population-based samples will be necessary to establish whether the DNA methylation signals identified here, also interact with genetic variants, or are subject to gene-environment interactions. We assessed whether our top-ranked results were associated with specific environmental disease risk factors, such as age and smoking. We therefore compared the most-associated pan-cancer signals to previously identified age and smoking methylation signals, but found no evidence that risk factors impact the identified cancer DMPs. Therefore the changes that we have detected are not simply biomarkers of these cancer risk factors. Whereas the BMI difference within a twin pair was not correlated with cancer incidence of the twins, three out of the 41 pairs had greater BMI in the twin diagnosed with cancer who was classified as obese within the BMI range of 30–40 kg/m^2^. However, the very small sample suggests that this concordance may have a negligent or very small effect on the results.

A MZ disease-discordant twin design of 41 twin-pairs in the discovery stage results in good power to detect moderate changes in DNA methylation. This study design is especially powerful in detecting differences in DNA methylation relatively independent of genetic variation with the need of fewer samples. On the other hand, the use of a surrogate tissue and the exploration of such a heterogeneous phenotype may reduce study power [63]. We estimate that we had 56 % power to detect the effect size of our top ranked probe at a conservative Bonferroni cut-off with the available sample [64], and would require 98 discordant twin pairs to reach 80 % power. The direct replication sample consisted of only 9 discordant MZ pairs of mixed sex, which provide low (10 %) mean power to detect the differential methylation effect observed at the top-ranked signal at nominal significance, and no power at a Bonferroni cut-off. However, access to these rare samples, can still give us an indication whether similar effects are observed in an independent dataset.

## Conclusions

In conclusion, this is the first pan-cancer analysis of cancer-discordant twins using blood samples collected up to 5 years before the diagnosis. In this MZ cancer-discordant study, we identified one novel significant pan-cancer signal and three suggestive results in whole blood samples. The top-ranked pan-cancer signal was upstream of a known tumour suppressor gene, and the methylation effect was observed prior to diagnosis, making it a strong blood-based cancer biomarker candidate. Altogether, three of the four DNA methylation signals exhibited differential methylation effects prior to cancer diagnosis, and show potential, if can be robustly replicated by others, to have clinical utility as pan-cancer biomarkers.

## Methods

### Sample selection

Detailed information regarding cancer diagnosis was obtained from the National Cancer Registry at the Office for National Statistics (ONS) for twin-pairs registered with the TwinsUK Adult Twin Registry. Written informed consent from all subjects was obtained in accordance with Guy’s & St Thomas’ NHS Foundation Trust Ethics Committee (EC04/015—15-Mar-04). The twins in this registry were similar in means and ranges of quantitative phenotypes from an age-matched population in the UK [65]. DNA methylation data for 41 middle-aged (age range 42–79, median age 61 years old) female MZ twin-pairs of European descent were included in the study. Discordant MZ twin-pairs were selected based on the criteria that one twin was diagnosed at least once with malignant tumour development of a solid organ while the co-twin was never diagnosed with malignant tumour development to date, in the period ranging from 4 to 21 years after cancer diagnosis of the co-twin (median = 10.3 years). In total, cancers at eight different primary locations were included: the breast (23 pairs), cervix (1 pair), colon (10 pairs), endometrium (1 pair), thyroid gland (1 pair), melanoma (3 pairs), ovary (1 pair), and pancreas (1 pair). Whole blood samples for DNA methylation profiling were obtained within 5-years prior to diagnosis (15 pairs) and post diagnosis (26 pairs). Samples were excluded if individuals had blood and lymph system-related cancers, skin cancer except melanoma (i.e., basal cell carcinoma and squamous cell carcinoma), cervical cancers except adenocarcinoma, as well as blood samples obtained outside the 5-year window surrounding cancer diagnosis for the main study.

The 41 cancer-discordant MZ twin-pairs were assessed for discordance in body mass index (BMI), alcohol intake, and smoking, which are considered to be major risk factors for cancer development. The mean BMI across all subjects was 26.9, and 21 out of 41 pairs had a greater BMI in the cancer-affected twin than in the unaffected co-twin. The mean BMI within-pair difference was 1.6 kg/m^2^, with three pairs that had a difference greater than 6 kg/m^2^ concordant with cancer status. Self-reported alcohol intake did not differ significantly within twin-pairs. In terms of smoking habits, 29 MZ twin-pairs were concordant: 19 MZ twin-pairs were non-smokers, 1 MZ twin-pair was current smoker, and 9 MZ twin-pairs were ex-smokers (stopped smoking at least 3 years before blood sample collection). The smoking-discordant 12 MZ twin-pairs comprised of 7 MZ twin-pairs including an ex-smoker and non-smoker co-twin, and 5 MZ twin-pairs including an ex-smoker and current smoker co-twin.

To assess the biomarker potential of methylation signals, one follow-up analysis included five additional MZ twin-pairs where blood samples were obtained 5–11 years prior to the diagnosis only. The additional five cancer-discordant MZ twin-pairs (age range 38–62, median age 57) were discordant for cancers at two different primary locations, the breast (3 pairs) and colon (2 pairs). There was no discordance in smoking or alcohol intake habits. The BMI of these individuals ranged from 20.5 to 27.8 kg/m^2^, and the median BMI within-pair difference was 0.5 kg/m^2^.

### Genome-wide DNA methylation data

Genome-wide DNA methylation profiles were obtained from 92 bisulfite-converted DNA whole blood samples, assayed by Illumina Infinium HumanMethylation450 BeadChip in two batches of 24 and 68 samples. The Infinium array targets 485,764 CpG sites across the genome and quantifies DNA methylation levels at a single CpG resolution as beta values, denoting the ratio of intensity signal from the methylated probes over the sum of intensity signals from both unmethylated and methylated probes, resulting in a beta value between 0 (unmethylated) and 1 (methylated). The probes cover 99 % of RefSeq genes and are distributed across the genome in the following manner: 20.75 % in promoter regions, 5 % in 5′ untranslated regions (UTR), 32.30 % in gene bodies, 3 % in 3′ UTR, and 24.60 % intergenic regions; and 14 % of CpG-sites not near genes in the genome [66].

Pre-processing consisted of five initial stages of quality control. First, three sets of probes were removed: probes that failed detection in at least one sample and with a bead count less than three in more than 5 % of the samples (*n* = 3325), probes for which the 50 bp sequence aligned to multiple locations in the genome (*n* = 17,764), and probes located on the sex chromosomes (*n* = 11,650) [67]. The remaining number of probes for analysis was 453,627. Second, the data was inspected visually for outliers using beta density plots, boxplots, and a heatmap of pair-wise correlation coefficients of genome-wide DNA methylation patterns. Third, MZ twin-pairs were verified using their known genotype and the 57 autosomal control SNP probes on the array. Fourth, 203 genomic imprinted regions were assessed using the R package wateRmelon [68], and no extreme outliers were detected. Lastly, probes were removed if they contained SNPs occurring in European populations with a frequency of >1 % and where the SNPs were located within 10 bp of the probe CpG (*n* = 17,544) [69]. The beta values were normalised using the BMIQ method to correct for probe type bias [70].

Principal component analysis (PCA) was performed on normalised beta values (*N*(0,1) at each probe. The first five principle components (PCs) combined explained 46.8 % of the total variance and were tested for associations with cancer status and likely covariates for DNA methylation data including array, position on the array, age, batch, and blood cell counts (CD4+ T cells, CD8+ T cells, granulocytes, monocytes, natural killer (NK) cells, and B cells) estimated using a published algorithm [71]. No association between cancer status and the first five PCs could be detected; however, significant associations (*P* < 4.1 × 10^−3^) with CD8+ T cells, CD4+ T cells, natural killer cells, granulocytes, monocytes, batch, and array were identified.

### Gene expression profiles

Gene expression profiles of the lymphoblastoid cell lines (LCLs), skin tissue, and adipose tissue from 283 healthy female individuals of European descent of the TwinsUK Adult Registry were obtained from the Multiple Tissue Human Expression Resource (MuTHER) project as previously described [72]. In short, punch biopsies (8 mm) were taken from a photo-protected area adjacent and inferior to the umbilicus of which the subcutaneous adipose tissue and skin tissue were dissected. LCLs were generated through EBV-mediated transformation of B-lymphocytes from peripheral blood samples collected at the same time point. RNA was extracted and expression profiling was performed using Illumina Human HT-12 v3 BeadChips. Probes with less than three beads present were excluded and log_2_-transformed expression signals were normalised separately per tissue, with quantile normalisation of the replicates of each individual followed by quantile normalisation across all individuals. Gene expression follow-up at each individual CpG site from the methylation analysis was performed using the nearest Illumina expression probe.

Whole blood DNA methylation in the same 283 healthy female individuals of European descent from TwinsUK was profiled using Illumina Infinium HumanMethylation450 BeadChip. Blood methylation data processing and quality control was performed as previously described.

### Statistical analysis

Analysis of global DNA methylation variation was performed using unsupervised hierarchical clustering analysis with Euclidean distances and complete linkage method. Within twin-pair correlations where assessed for all CpG sites between discordant MZ twin-pairs and as individuals randomly assigned to one of 41 pairs (permuted 100 times) either within disease status or independent of disease status. Correlation was assessed using a Spearman’s rank test. A two sample *t* test was performed to test for significant differences in correlation between the groups.

To identify differentially methylated positions (DMPs) associated with cancer discordance, a linear model was fitted on the normalised beta values per probe and the first five PCs. The PCs capture not only known variance due to technical covariates, such as array and batch, but also variability introduced by the mixture of different blood cell types present in whole blood samples. This method is similar to reference-free computational approaches applied in recent published methods to control for cell heterogeneity and noise in large-scale datasets [73, 74]. The difference of DNA methylation residuals was calculated within twin-pairs in consistent direction (cancer-affected twin − healthy twin) and was followed by a one-sample *t* test on these data. Results were considered significant if they surpassed a false discovery rate (FDR) threshold of 10 %, estimated using qvalue in *R*. Suggestive results were considered if they surpassed nominal *P* value of 1 × 10^−5^. The top results were compared to DMP analyses using different methods for adjusting for blood cell types and technical covariates, including the computational approach ‘RefFreeEWAS’ (using PairsBootRefFreeEwasModel) [73] and an approach using the covariates identified by PCA earlier described (batch, granulocytes, CD8 T cells, and NK cells). The top-ranked probe, ranked first in all three methods, and the suggestive probes had *P* values <1 × 10^−03^ across the three approaches.

Differentially methylated regions (DMRs) associated with cancer discordance were identified using R package ‘bumphunter’ [40]. Regions were identified of at least three consecutive probes with a maximum gap of 500 bp between the locations of each probe. The difference in adjusted DNA methylation values (described above for DMP analyses) was calculated within twin-pairs in consistent direction (cancer-affected twin − healthy twin) and was compared to a group without DNA methylation differences using bumphunter. The cut-off used was set at 0.6 with 1000 permutations with potential DMRs identified with a *P* value <0.05 and family-wise error rate (FWER) *P* value <0.5, as estimated within bumphunter [40].

To assess whether pan-cancer DMPs were also likely to be associated with environmental cancer risk factors such as age and smoking, we selected previously published DMPs for age [44] and smoking [8] in whole blood. We then counted the occurrence of age or smoking DMPs within the top 500 ranked cancer DMP probes, and in the remainder of the probes in this study (453,127). Enrichment was assessed using Fisher’s exact test.

Gene expression analysis at genes in or near the top-ranked DNA methylation probes was performed in a sample of 283 healthy individuals in multiple tissues. A linear mixed-effects model was fitted on the expression data with age, BMI, batch, concentration (skin tissue only) as fixed effects, and family and zygosity as random effects. A similar linear mixed-effects model was fitted on the blood DNA methylation data in these individuals with age, BMI, array, position on the array, and granulocytes, monocytes, CD8 T cells (estimated) as fixed effects, and family and zygosity as random effects. Residuals from both models were compared with Pearson correlation.

### Replication

Replication was pursued in MZ twin pairs registered with the Netherlands Twin Register (NTR). Detailed information regarding cancer diagnosis was obtained through record linkage with the Netherlands Cancer Registry (NKR). DNA methylation data and white blood cell counts were available for 703 MZ pairs who took part in the NTR biobank project [75] of which 15 were identified as discordant for any type of cancer. Discordant MZ twin-pairs for replication were selected based on the criteria that one twin was diagnosed at least once with malignant tumour development while the co-twin was never diagnosed with malignant tumour development to date. After excluding pairs with blood samples collected >5 years after the cancer diagnosis and pair discordant for Hodgkin’s disease, mature B cell or cervical cancer, nine middle-aged (age range 35–72, median age 52 years old) MZ twin-pairs were selected for the replication analysis, and these were four male and five female MZ twin-pairs. In total, cancers at six different primary locations were included: the breast (3 pairs), meninges (1 pair), pituitary gland (1 pair), prostate (2 pairs), rectum (1 pair), and soft tissue (1 pair).

Direct replication of the findings at the four top-ranked signals was pursued in the nine NTR cancer-discordant MZ pairs using the same quality control, normalisation, and analysis pipeline as the original discovery analysis in 41 MZ pairs. In addition, we also assessed whether cancer-discordant twins showed greater methylation variability compared to healthy concordant MZ twins at the top-ranked cancer probes. The variability analysis was performed on normalised (functional normalisation [76]) betas at three of the four top-ranked probes available in the overall NTR dataset, comparing 9 cancer-discordant to 480 healthy concordant MZ twin-pairs for which blood samples were obtained on the same time point. In the variability analysis, absolute within-pair methylation differences were determined of the 480 healthy MZ twin-pairs as well as the within-pair differences of the 9 cancer-discordant MZ twin-pairs and assessed significance by a Mann-Whitney *U* test.

### Genomic annotation analysis

Significant and suggestive results were annotated with respect to defined CpG islands (CpG Islands [CGI], shores, shelves) and relative to RefSeq genes (promoter, 5′UTR end, gene body, 3′UTR, intergenic). The top-ranked results were further explored using data generated from the ENCODE project for histone modification signatures used in ChromHMM state analysis, DNase I hypersensitivity sites, and transcription factor binding sites.

Enrichment of functional genomic elements was performed at the top 500 pan-cancer-ranked DMP probes, compared to the remainder of the probes in this study (453,127). Enrichment was performed with respect to the majority of annotation categories described above. We used ChromHMM classifications at 15 chromatin states of the GM12878 cell line from ENCODE data [77, 78]. Analyses were initially performed over 15 states, and subsequently, we also merged states active promoter (state 1), weak promoter (state 2), and poised promoter (state 3) were into a single ‘promoter’ category, and states strong enhancer (states 4 and 5), and weak enhancer (states 6 and 7) into a single ‘enhancer’ category. Enrichment tests were performed in the merged and individual categories, and nominally significant results were obtained for state weak enhancer (state 7) and merged enhancer category. Fisher’s exact test was used to assess significance.

### Availability of supporting data

The dataset will be uploaded to GEO.

## Additional files

Additional file 1: Figure S1. Pan-cancer top-ranked results in 41 discordant MZ twin-pairs. Direction of association at **[A]** cg26079695 in *COL11A2*, **[B]** cg27094856 in *AXL*, and **[C]** cg21046959 in *LINC00340*. Results are plotted using normalised unadjusted beta values of cancer-affected individuals (left) and healthy individuals (right). The lines connect co-twins in twin-pairs and indicate a consistent direction of effect. (PDF 78 kb) Additional file 2: Figure S1. Replication of four top-ranked pan-cancer DMPs in 9 cancer discordant MZ twin-pairs. Direction of association at **[A]** cg0244695 near *SASH1*, **[B]** cg26079695 in *COL11A2*, **[C]** cg27094856 in *AXL*, and **[D]** cg21046959 in *LINC00340*. Results are plotted using normalised unadjusted beta values of cancer-affected individuals (left) and healthy individuals (right). The lines connect co-twins in twin-pairs and indicate a consistent direction of effect. (PDF 88 kb) Additional file 3: Figure S1. Variability at three top-ranked pan-cancer DMPs in 9 cancer discordant and 480 healthy MZ twin-pairs. Histogram with density overlay of absolute differences of 480 healthy MZ twin-pairs with median (blue) and density and median of differences between 9 cancer discordant MZ twin-pairs (red). At **[A]** cg0244695 near *SASH1*, **[B]** cg26079695 in *COL11A2*, and **[C]** cg27094856 in *AXL*. (PDF 143 kb) Additional file 4: Table S1. Top-ranked results from EWAS of 15 cancer discordant MZ twin-pairs prior to diagnosis. (XLSX 42 kb) Additional file 5: Figure S1. Differential methylation with respect to age and time of cancer diagnosis. **[A]** Time to diagnosis compared to age at blood sample collection, the line represent the least squares regression fit. **[B]** Unadjusted DNA methylation values at cg02444695 (near *SASH1*) in affected individuals (red) and healthy co-twins (blue), shown with respect to age at blood sample collection (years) with smooth (LOESS) lines fitted for both groups. **[C–F]** Unadjusted DNA methylation values in affected individuals (red) and healthy co-twins (blue), shown with respect to time of diagnosis (years) with smooth (LOESS) lines fitted for both groups with blood samples collected 5 to 11 years before cancer diagnosis. The orange and black vertical lines represent the time of diagnosis and time window of the main study respectively. At **[C]** cg0244695 near *SASH1,* **[D]** cg26079695 in *COL11A2,* **[E]** cg27094856 in *AXL*, and **[F]** cg21046959 in *LINC00340*. (PDF 185 kb)
