# Supplementary figures and images for: Integrative DNA methylome analysis of pan-cancer biomarkers in cancer discordant monozygotic twin-pairs
Source: Clin Epigenetics. 2016 Jan 20;8:7. doi: 10.1186/s13148-016-0172-y (PMC4721070; doi:10.1186/s13148-016-0172-y)

Figure S1

**A**

cg26079695 in *COL11A2*

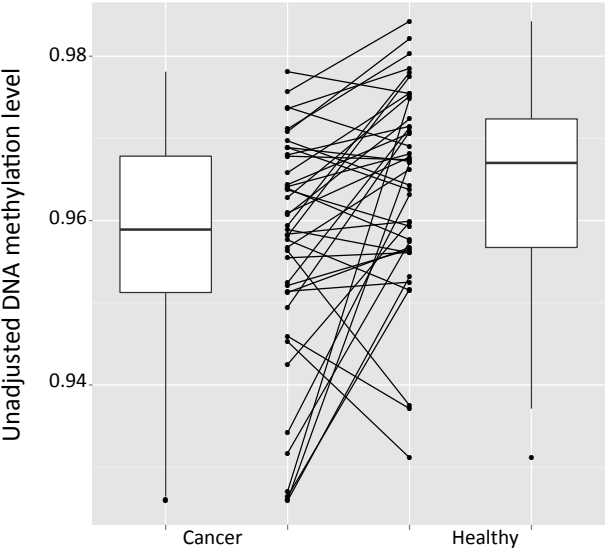

**B**

cg27094856 in *AXL*

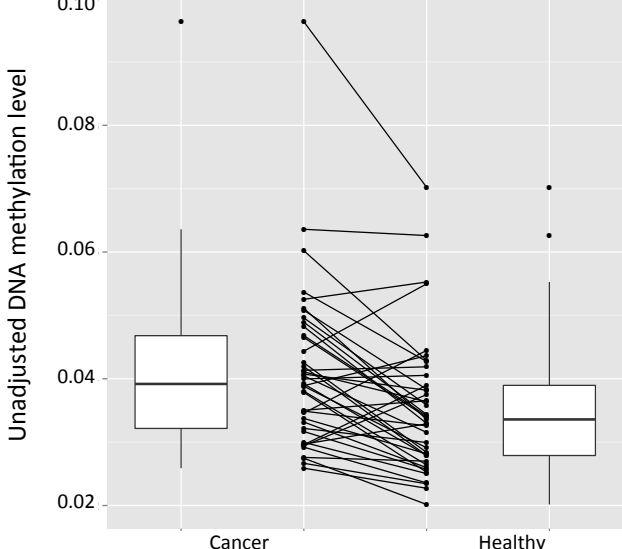

**C**

cg21046959 in *LINC00340*

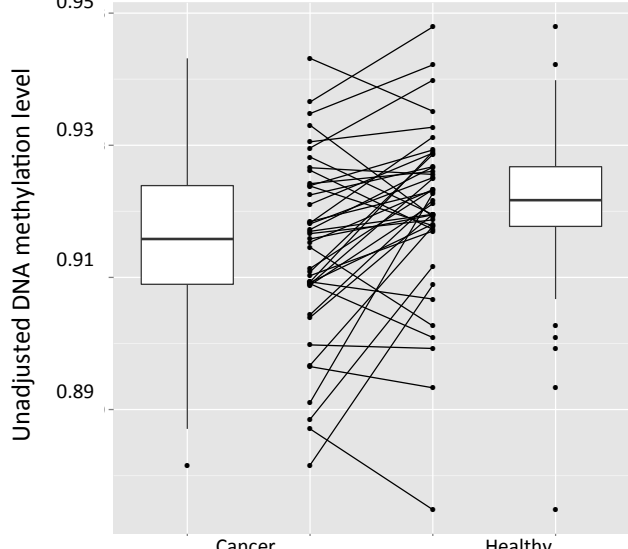

Supplement: Additional file 1: Figure S1. — Pan-cancer top-ranked results in 41 discordant MZ twin-pairs. Direction of association at [A] cg26079695 in COL11A2, [B] cg27094856 in AXL, and [C] cg21046959 in LINC00340. Results are plotted using normalised unadjusted beta values of cancer-affected individuals (left) and healthy individuals (right). The lines connect co-twins in twin-pairs and indicate a consistent direction of effect. (PDF 78 kb) [file 13148_2016_172_MOESM1_ESM.pdf]

Figure S1

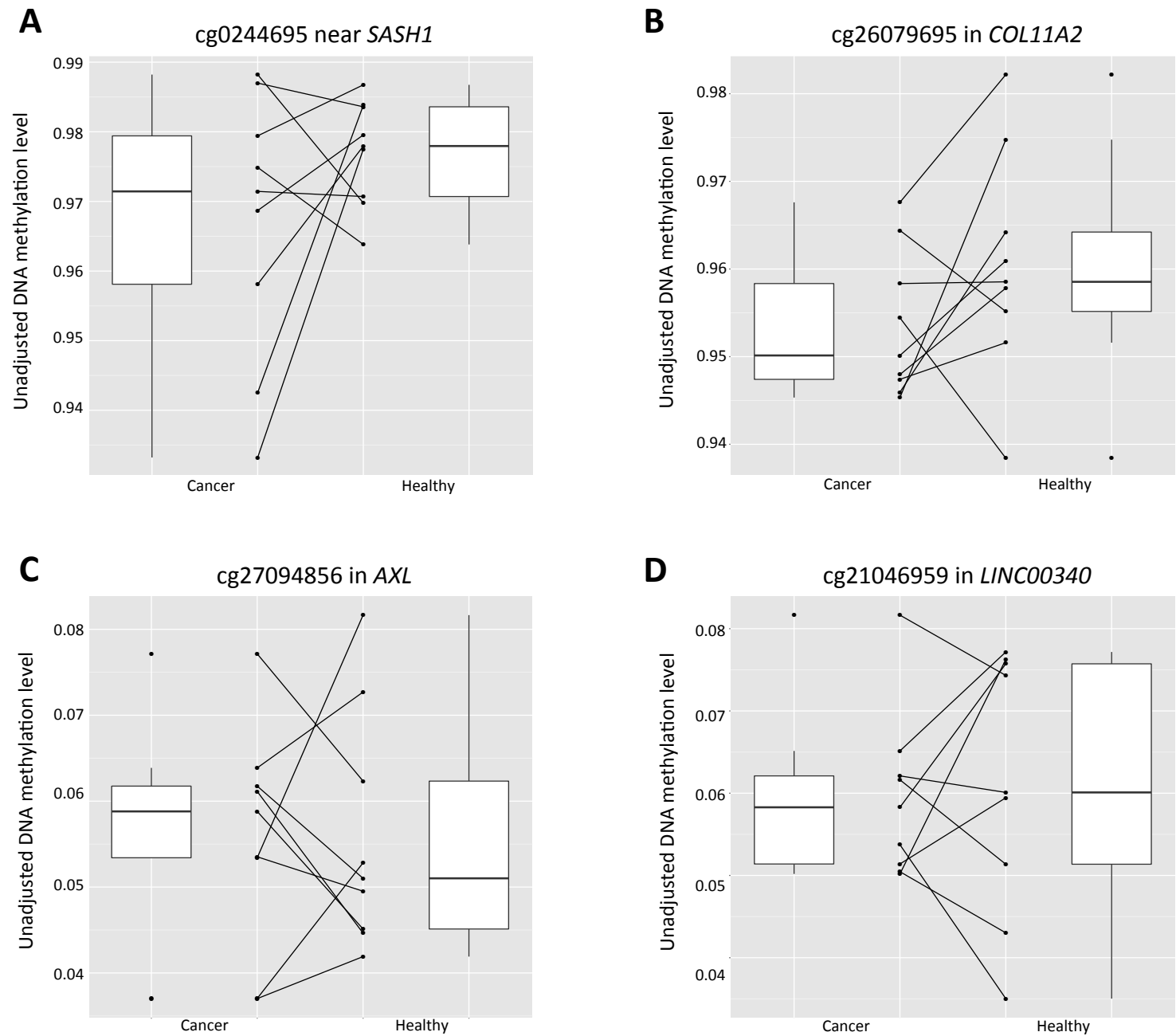

Supplement: Additional file 2: Figure S1. — Replication of four top-ranked pan-cancer DMPs in 9 cancer discordant MZ twin-pairs. Direction of association at [A] cg0244695 near SASH1, [B] cg26079695 in COL11A2, [C] cg27094856 in AXL, and [D] cg21046959 in LINC00340. Results are plotted using normalised unadjusted beta values of cancer-affected individuals (left) and healthy individuals (right). The lines connect co-twins in twin-pairs and indicate a consistent direction of effect. (PDF 88 kb) [file 13148_2016_172_MOESM2_ESM.pdf]

Figure S1

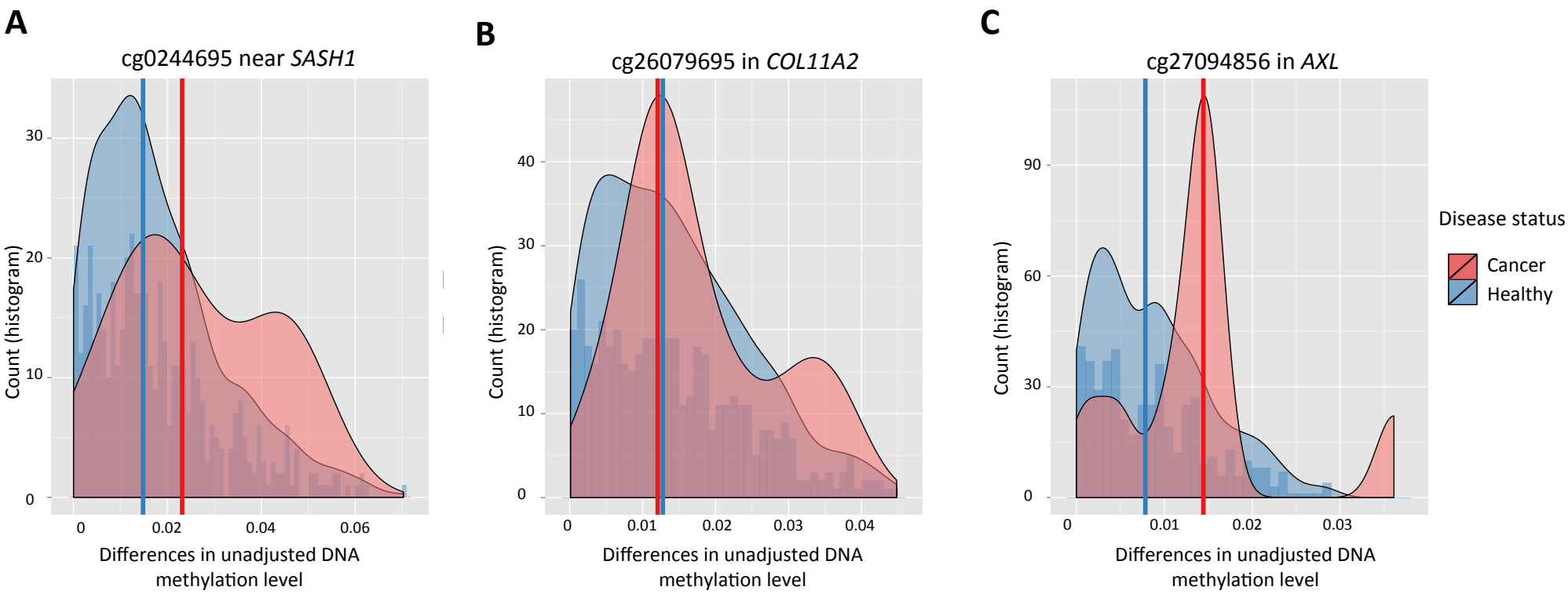

Supplement: Additional file 3: Figure S1. — Variability at three top-ranked pan-cancer DMPs in 9 cancer discordant and 480 healthy MZ twin-pairs. Histogram with density overlay of absolute differences of 480 healthy MZ twin-pairs with median (blue) and density and median of differences between 9 cancer discordant MZ twin-pairs (red). At [A] cg0244695 near SASH1, [B] cg26079695 in COL11A2, and [C] cg27094856 in AXL. (PDF 143 kb) [file 13148_2016_172_MOESM3_ESM.pdf]

Figure S1

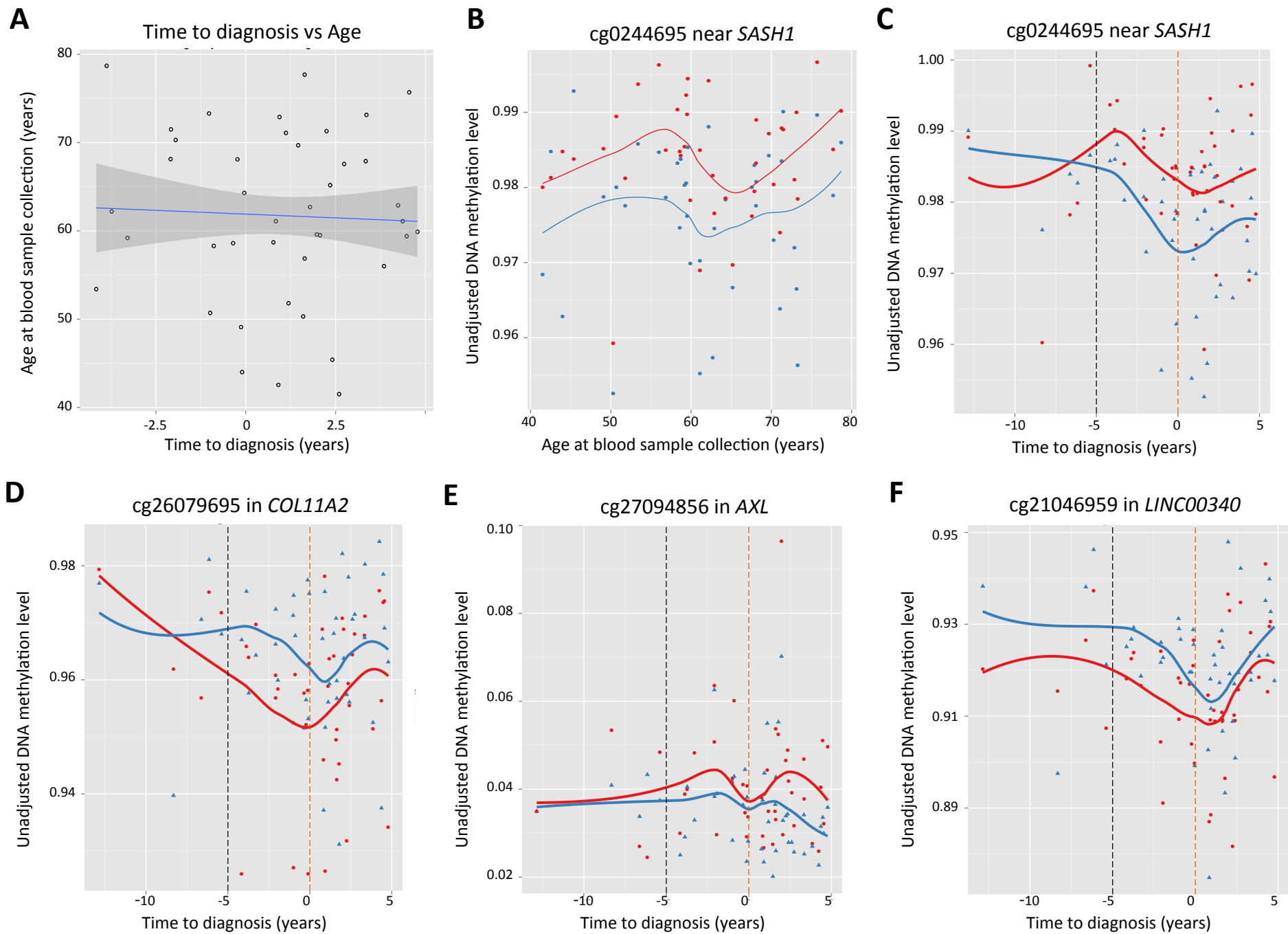

Supplement: Additional file 5: Figure S1. — Differential methylation with respect to age and time of cancer diagnosis. [A] Time to diagnosis compared to age at blood sample collection, the line represent the least squares regression fit. [B] Unadjusted DNA methylation values at cg02444695 (near SASH1) in affected individuals (red) and healthy co-twins (blue), shown with respect to age at blood sample collection (years) with smooth (LOESS) lines fitted for both groups. [C–F] Unadjusted DNA methylation values in affected individuals (red) and healthy co-twins (blue), shown with respect to time of diagnosis (years) with smooth (LOESS) lines fitted for both groups with blood samples collected 5 to 11 years before cancer diagnosis. The orange and black vertical lines represent the time of diagnosis and time window of the main study respectively. At [C] cg0244695 near SASH1, [D] cg26079695 in COL11A2, [E] cg27094856 in AXL, and [F] cg21046959 in LINC00340. (PDF 185 kb) [file 13148_2016_172_MOESM5_ESM.pdf]
